# *Syndecan-4* as a genetic determinant of the metabolic syndrome

**DOI:** 10.1186/s13098-023-01132-8

**Published:** 2023-07-17

**Authors:** Paolina Crocco, Denise Vecchie, Sreejit Gopalkrishna, Serena Dato, Giuseppe Passarino, Martin E. Young, Prabhakara R. Nagareddy, Giuseppina Rose, Maria De Luca

**Affiliations:** 1 Department of Biology, Ecology, and Earth Sciences, University of Calabria, Rende, 87036 Italy; 2 Department of Nutrition Sciences, University of Alabama at Birmingham, Birmingham, AL 35294 USA; 3 Department of Surgery, Division of Cardiac Surgery, The Ohio State University Wexner Medical Center, Columbus, OH USA; 4 Division of Cardiovascular Diseases, Department of Medicine, University of Alabama at Birmingham, Birmingham, AL 35294 USA

**Keywords:** Heparan sulfate proteoglycans, Obesity, Lipid profile, Insulin resistance, Metabolic syndrome, Single nucleotide polymorphism

## Abstract

**Background:**

Syndecan-4 (SDC4) is a member of the heparan sulfate proteoglycan family of cell-surface receptors. We and others previously reported that variation in the *SDC4* gene was associated with several components of the metabolic syndrome, including intra-abdominal fat, fasting glucose and triglyceride levels, and hypertension, in human cohorts. Additionally, we demonstrated that high fat diet (HFD)-induced obese female mice with a *Sdc4* genetic deletion had higher visceral adiposity and a worse metabolic profile than control mice. Here, we aimed to first investigate whether the mouse *Sdc4* null mutation impacts metabolic phenotypes in a sex- and diet-dependent manner. We then tested whether *SDC4* polymorphisms are related to the metabolic syndrome (MetS) in humans.

**Methods:**

For the mouse experiment, *Sdc4-*deficient (*Sdc4*^*−/−*^) and wild-type (WT) mice were treated with 14-weeks of low-fat diet (LFD). Body composition, energy balance, and selected metabolic phenotypes were assessed. For the human genetic study, we used logistic regression models to test 11 *SDC4* SNPs for association with the MetS and its components in a cohort of 274 (113 with MetS) elderly subjects from southern Italy.

**Results:**

Following the dietary intervention in mice, we observed that the effects of the *Sdc4* null mutation on several phenotypes were different from those previously reported in the mice kept on an HFD. Nonetheless, LFD-fed female *Sdc4*^*−/−*^ mice, but not males, displayed higher levels of triglycerides and lower insulin sensitivity at fasting than WT mice, as seen earlier in the HFD conditions. In the parallel human study, we found that carriers of *SDC4* rs2228384 allele C and rs2072785 allele T had reduced risk of MetS. The opposite was true for carriers of the *SDC4* rs1981429 allele G. Additionally, the SNPs were found related to fasting triglyceride levels and triglyceride glucose (TyG) index, a reliable indicator of insulin resistance, with sex-stratified analysis detecting the association of rs1981429 with these phenotypes only in females.

**Conclusions:**

Altogether, our results suggest that *SDC4* is an evolutionary conserved genetic determinant of MetS and that its genetic variation is associated with fasting triglyceride levels in a female-specific manner.

**Supplementary Information:**

The online version contains supplementary material available at 10.1186/s13098-023-01132-8.

## Introduction

Metabolic syndrome (MetS) is a cluster of inter-related and partly heritable metabolic disorders that include abdominal obesity, hyperglycemia, dyslipidemia, and hypertension [1, 2]. Together these factors dramatically increase the risk of developing atherosclerotic cardiovascular disease, insulin resistance, and diabetes mellitus [3, 4], as well as premature death [5, 6]. Given its adverse outcomes and high prevalence worldwide, ranging from 12.5 to 31.4% [7], MetS has become a serious global health issue that is likely to get worse in the next decades due to population aging [8].

The mammalian syndecan (SDC) family of heparan sulfate proteoglycans (HSPG) consists of four transmembrane proteins (SDC1-SDC4), each encoded by separate genes [9], which are involved in several developmental and disease processes [10–13]. SDC4 is the only member of the SDC family with almost ubiquitous distribution [9] and the only one that promotes focal adhesion assembly around pre-existing integrin clusters on fibronectin [14]. Like the other SDCs, SDC4 is characterized by an extracellular domain (ectodomain) with attachment sites for glycosaminoglycan chains that mediate interactions with a wide array of ligands, including soluble growth factors, morphogens, cytokines, and lipoprotein lipase (LPL) [11, 15, 16]. The ectodomain is followed by a highly conserved transmembrane domain and a short cytoplasmic tail, which, in turn, consists of two highly conserved regions separated by a variable region that is specific to each SDC [17]. It is through a PDZ-binding motif localized in the variable region that SDC4 regulates several cellular processes, including adaptive cell stiffness in pluripotent stem cells [18] and subcellular localization of mechanistic target of rapamycin (mTOR) Complex 2 and Akt activation in endothelial cells [19].

Over the past few years, it has become clear that SDC4 plays a crucial role in the function of major metabolic tissues/organs, such as adipose tissue [20], liver [21, 22], and skeletal and cardiac muscles [23, 24]. In this regard, we and others reported that variants in the *SDC4* gene correlate with MetS endophenotypes [25–27]. Specifically, we demonstrated that three *SDC4* single nucleotide polymorphism (SNPs) were associated with inter-individual variability in fasting glucose levels (rs4599), insulin sensitivity (rs2267871), resting energy expenditure (REE) (rs4599), and intra-abdominal fat (rs1981429) in a cohort of American peripubertal children [25]. The latter finding is perhaps the most notable since we showed that the minor G-allele of rs1981429 was significantly associated not only to more intra-abdominal fat in American children [25], but also to higher levels of fasting plasma triglycerides (TG) in healthy elderly Italian subjects (age 64 to 107 years), in a follow-up study [26]. Furthermore, in an independent work from Kunnas & Nikkari [27], rs1981429 was found to be linked with hypertension and increased prevalence of coronary artery disease (CAD) in a cohort of middle-aged (45–50-year-old) subjects from the Tampere adult population cardiovascular risk study. In this latter study, the *SDC4* rs1981429 T-allele was found associated with hypertension and CAD risk instead of the G-allele [27]. Yet, this result is not surprising considering that gene-by-environment interactions modulate cardiovascular risk factors [28] and Finnish people have been exposed to different physical and social environmental factors than Italians [29, 30].

Our genetic findings in humans were substantiated by a study that we performed in mice harboring a *Sdc4* global knockout allele [20]. After inducing obesity in *Sdc4-*deficient (*Sdc4*^*−/−*^) and wild-type (WT) mice by feeding the animals a high-fat diet (HFD), we observed that female *Sdc4*^*−/−*^ mice, but not males, displayed increased adiposity, higher fasting levels of plasma total cholesterol (TC), triglyceride (TG), and glucose, and lower insulin sensitivity than WT [20]. Altogether, our earlier work strongly suggests that *SDC4* is an evolutionary conserved genetic determinant of the MetS, an idea that we sought to further evaluate in the current study.

In mice, like in humans, genetic effects at pleiotropic loci associated with normal variation in several MetS components are often context-dependent, differing between sexes, as seen in the *Sdc4*^*−/−*^ mice kept on an HFD, and depending on environmental parameters, such as dietary patterns [31]. Thus, in this study we first assessed whether the effects of the mouse *Sdc4* null mutation on body composition, energy balance, and selected metabolic phenotypes might be influenced by the composition of the diet. Subsequently, we investigated whether *SDC4* variation is related to human MetS and its components in a cohort of 274 elderly subjects, of whom 113 were diagnosed with MetS.

## Materials and methods

### Animal experiments

As previously reported [20], *Sdc4*^*−/−*^ mice were provided by the Geir Christensen lab at the University of Oslo after being repeatedly backcrossed to a C57BL/6J inbred background. Seven-week-old female and male *Sdc4*^*−/−*^ and WT C57BL/6J mice were maintained on a diet that was low in fat (10% kcal fat), but high in carbohydrates (70% kcal carbohydrate) (D12450B, Research Diets Inc., New Brunswick, NJ USA) for 14 weeks. Mice were kept in groups (up to seven per cage) in a housing room set to a 12-h light/dark cycle, (with Zeitgeber Time (ZT) 12 at the start of the dark phase), a controlled- temperature of 22 °C, and a relative humidity of about 50%. Animals were given *ad libitum* access to food and water, except when fasting blood specimens were obtained. Body weight was recorded at baseline and weekly for the first 7 weeks and then at weeks 11, 12, and 14.

Measures of body composition and energy balance phenotypes in conscious mice were performed at the UAB Small Animal Physiology Core using a noninvasive quantitative magnetic resonance imaging system (EchoMRI™ 3-in-1 v2.1; Echo Medical Systems, Houston, TX) and an 8-cage CaloSys indirect calorimetry system (TSE Systems, Inc., Chesterfield, MO), respectively, as previously described [20]. Briefly, after the mice were acclimated to individual metabolic cages for 48 h, O_2_ consumption, CO_2_ production, and spontaneous locomotor activity were continuously measured for 24 h. REE was determined as the average of the three lowest 18 min-intervals, with at least 1 h in between intervals. To minimize the weight loss during acclimation in most animals due to them not adjusting to the center-mounted food hopper of the metabolic cages, food was placed in both the cage and the hopper. Thus, only one food intake for each acclimation day was recorded and the average of the two days acclimation period was calculated. Percentage fat and lean mass relative to total body mass were calculated as [fat mass (or lean mass)/body weight] × 100.

Blood samples were collected after six hours of fasting that started at 7:00 am (ZT1). Plasma levels of TC and TG in plasma were measured using commercially available kits (FUJIFILM Wako Diagnostics USA Corporation, Richmond, VA). For the oral glucose tolerance test (OGTT), 25% of D-(+)-glucose was administered orally by gavage after six hours of fasting at a dose of 2 g/kg and blood samples were taken at 0, 15-, 30-, 60-, and 120-minutes post-glucose load. Blood glucose was measured directly from the tail tip using a One Touch Ultra 2 glucose monitoring system (Lifescan, Johnson & Johnson, USA). Plasma insulin was measured using an ELISA kit from ALPCO Immunoassays (Salem, NH) per manufacturer’s instructions. Whole-body insulin sensitivity index (ISI) was derived from the OGTT using the Matsuda & DeFronzo’s equation [32].

Following experimentation, all animals were euthanized, and the gonadal fat pads were excised and fixed in 10% neutral buffered formalin. Tissues were embedded in paraffin, sectioned, and stained with hematoxylin and eosin (H&E) at the UAB Comparative Pathology Laboratory. Digital images were acquired with a microscope Leica DMRB using a color camera and XnView software. Five medium-power field (20×) images were acquired at regular spatial intervals from three to four animals in each group. Adipocyte area was determined by measuring approximately 100 cells per animal using software Motic-Images Plus 2.0 [20].

### Statistics for animal study

A three-way analysis of variance (ANOVA) was performed to compare percent changes in body weight over the intervention period, with time, sex, and genotype and all possible interaction terms included in the model. Mauchly’s test was used to assess the assumption of sphericity and Greenhouse-Geisser correction was used for violation of this assumption.

Two-way ANOVA/ANCOVA models and repeated measures ANOVA were used, as appropriate, to investigate the effects of sex, genotype, and their interaction on body composition, energy balance, and metabolic phenotypes. Fat and lean mass were included as covariates in the model for REE. A log^10^ transformation was applied to the data that did not meet the assumption of normality. The Tukey test for *post-hoc* pairwise comparisons was implemented to assess significant differences between groups.

The Kolmorov’s D statistic implemented in SAS was used to test whether the cumulative relative frequency of epididymal adipocyte size in male *Sdc4*^*−/−*^ mice was different from WT mice.

Cosinor analyses were performed to determine whether 24 h time series data significantly fit a cosine curve; if they did, then mesor (daily average value), amplitude (peak-to-mesor difference), and acrophase (timing of the peak) were calculated and compared between genotypes, as described previously [33].

Statistical analyses were performed with SAS (v. 9.4), GraphPad Prism (v. 9.2.0) and SPSS (v. 29.0). A significant level of 0.05 was used throughout the animal study.

### Human study

DNA samples from 274 unrelated subjects with age ranging from 65 to 85 years were analyzed. Of these subjects, 113 were affected by MetS and 161 were healthy individuals who were matched for sex ratio (about 1:1 for both groups) and mean age with the affected group. All samples were collected within the framework of recruitment campaigns carried out for examining the quality of aging in Calabria region (southern Italy). Information on participants’ socio-demographic characteristics and health status, including medical history and medication use, was acquired using a standardized questionnaire and through medical visit and clinical examination. Fasting venous blood was collected from each subject for clinical and laboratory examination and for DNA extraction. The study was conducted according to the guidelines established in the Declaration of Helsinki and approved by the local Ethical Committee. Each subject signed an informed consent for the permission to collect blood samples and usage of register-based information for research purposes.

Each subject was measured for waist and hip circumference by trained nurses, and waist-to-hip ratio (WHR) was directly calculated. Height and weight were measured while subjects were dressed in light clothes and without shoes and the BMI was calculated as weight (kg) /height squared (m^2^). Systolic blood pressure (SBP) and diastolic blood pressure (DBP) were measured three times with a mercury sphygmomanometer on the right arm after a rest period of more than 30 min. The average of the three measurements was calculated and taken as the final BP value. Fasting plasma glucose, glycosylated haemoglobin (HbA1c), TC, LDL-cholesterol (LDL-C), HDL-cholesterol (HDL-C), and TG measurements were performed using standard protocols. We used the triglyceride glucose (TyG) index as a surrogate measure for insulin resistance. The TyG index was calculated as Ln (fasting triglycerides [mg/dL] x fasting plasma glucose [mg/dL]/2) [34].

In accordance with the National Cholesterol Education Program Adult Treatment Panel III (NCEP ATP III) criteria [35], MetS was diagnosed when at least three of five of the following alterations are present: abdominal obesity (waist circumference > 102 cm in men or > 88 cm in women); hypertension (arterial blood pressure ≥ 130/≥85 mmHg, or antihypertensive medication treatment, and/or a history of hypertension); hyperglycemia (fasting blood glucose ≥ 110 mg/dl, and/or treatment with medications for T2D); hypertriglyceridemia (fasting TG level ≥ 150 mg/dl, and/or drug treatment for elevated triglycerides); low HDL cholesterol (HDL cholesterol < 40 mg dL in men or < 50 mg dL in women).

Eleven SNPs within approximately 26 kb encompassing the entire *SDC4* gene and its 5′ and 3′ flanking regions were genotyped in all subjects included in the study. The SNPs were selected to capture much of the genetic variation in the gene and from previous studies in which significant genetic associations with MetS endophenotypes were reported.

Multiplex SNP genotyping was performed by PCR followed by primer extension and MALDI-TOF mass spectrometry using iPLEX Gold technology from Sequenom (Sequenom Inc., San Diego, CA, USA). Primers for PCR and single base extension were designed by Sequenom MassARRAY Assay Designer software (version 3). Standard procedures were used to amplify PCR products, and unincorporated nucleotides were deactivated with the shrimp alkaline phosphatase (SAP). A primer extension reaction was subsequently implemented using the mass extension primer and the terminator. The primer extension products were then desalted on resin and spotted onto the 384-element SpectroCHIP (Sequenom) for MALDI-TOF analysis using SpectroACQUIRE v3.3.1.3 (Sequenom). Spectra were analyzed using MassARRAY Typer v3.4 Software (Sequenom). For quality control, about 10% of the analyzed samples were re-genotyped to assess the reliability of the genotype identification protocols. Concordance among duplicates was > 99.8% for all SNPs polymorphisms. Additional quality control procedures include the exclusion of SNPs out of the Hardy–Weinberg equilibrium in controls (p-value < 0.05) or low genotyping success rates (< 90%).

### Statistics for human study

Continuous variables were presented as mean and standard deviation. The Kolmogorov-Smirnov test was used to evaluate the normality of data distribution. Differences between groups for continuous variables were determined by the independent-samples t-test or Mann-Whitney U test according to distribution characteristics. Categorical variables were expressed as percentage and compared using the chi-squared test (χ2) test. For each SNP, departure from Hardy–Weinberg equilibrium was assessed in controls using the χ2 test. Logistic regression models were applied to estimate the effect of genetic variables on MetS susceptibility, using age and sex as covariates. Three genetic models were assessed for each SNP: additive, dominant, and recessive. The most likely genetic model was estimated based on minimum level of statistical significance (Wald’ test *p*-value). Only the dominant model was considered where the minor allele homozygote count for either cases or controls was lower than 3%. Linear regression models were applied to capture the effect of polymorphisms on clinical phenotype variables, with age and sex as confounding covariates. Where appropriate, variable levels were logarithmically transformed prior to statistical analysis to adhere to a normality assumption.

Linkage disequilibrium (LD) and haplotype analysis was performed using the SHEsis software [36]. The LDlink’s LDproxy tool [37] was used to identify potential proxy SNPs (CEU; r^2^ > 0.8).

Statistical analyses were carried out using SPSS software version 28.0 (SPSS, Inc., Chicago, IL, USA). All statistical tests were two-sided, and *p* < 0.05 was defined as statistically significant.

## Results

### Metabolic effects of *Sdc4* deficiency in LFD-fed mice

**Fig. 1:**
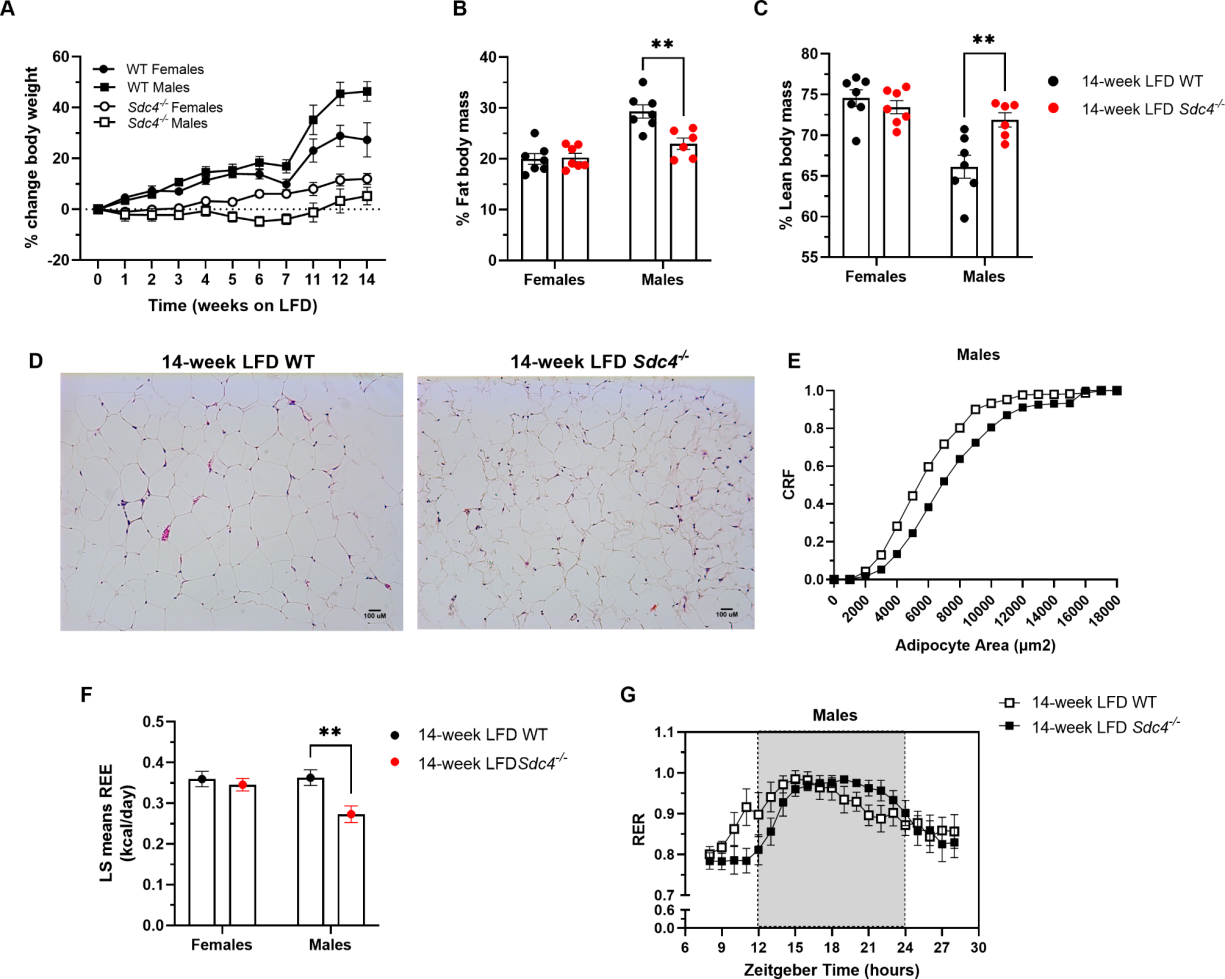
Sex-dependent alterations in body composition and energy expenditure in *Sdc4* deficient mice fed a 14-week LFD. **(A)** Under LFD conditions, male *Sdc4*^*−/−*^ mice did not show a statistically significant gain weight (Time*genotype*sex: F_10,230_=5.01, *p* < 0.0001). Values represent the mean of % change in body weights from baseline for *n* = 6–7 independent replicates. Error bars represent standard errors (SE). **(B-C)** Following the dietary intervention, male *Sdc4*^*−/−*^ mice displayed significantly less % fat body mass (panel B) and higher % lean body mass (panel C). Box and whiskers plots denote individual data points separated by a line representing the group median. Each individual value is plotted as a dot superimposed on the boxplots. In both panels, **p < 0.01 was obtained from Tukey *post hoc* tests for multiple comparisons. (D-E) LFD-fed male *Sdc4*^*−/−*^ mice had smaller adipocytes in epididymal WAT than WT mice. Representative H&E of epididymal WAT sections (panel D) and cumulative relative frequencies (CRF) distribution of adipocyte size (panel E) from male mice (100 cells per animal; *n* = 3–4). (F) LFD-fed male *Sdc4*^*−/−*^ mice exhibited lower fat- and lean-adjusted REE than male WT mice. Data indicates least-square means ± SE for *n* = 6–7 independent replicates. (G) LFD-fed male *Sdc4*^*−/−*^ mice displayed significantly different 24-hr fluctuations in RER than male WT mice. Data indicates mean ± SE. *n* = 6 animals per genotype

We observed a significant weight gain for both female and male WT mice during the fourteen-week LFD-feeding. However, only subtle changes in body weight were observed in *Sdc4*^*−/−*^ mice weight during the experimental period (Fig. 1A and Supplementary Table 1), suggesting that *Sdc4*-deficient mice are resistant to weight gain when fed a diet containing low fat content. This effect was observed in both sexes but was more robust in males, as revealed by a statistically significant interaction of time spent on the diet with genotype and sex (Time*genotype*sex: F_10,230_=5.01, *p* < 0.0001). Following the LFD treatment period, we also found that the *Sdc4* mutation affects percentage fat body mass (%FBM) and lean body mass (%LBM) but it does so in a sex-dependent manner (Genotype*sex: %FBM F_1,23_=9.32, *p* = 0.0056; %LBM F_1,23_=10.29, *p* = 0.0039) (Supplementary Table 2). As shown in Fig. 1, while females did not show any change in body composition, male *Sdc4*^*−/−*^ mice had 22% less %FBM than WT (Fig. 1B) and were 8% leaner (Fig. 1C). Consistently, histological examination of the gWAT (Fig. 1D) revealed that the adipocytes of male *Sdc4*^*−/−*^ mice were significantly smaller (D = 0.20, *p* = 0.0479) than male WT mice (Fig. 1E).

Placement of mice in metabolic cages revealed a sex-specific effect of *Sdc4* deficiency on fat- and lean-adjusted REE (F_1,21_=8.35, *p* = 0.0088), with male *Sdc4*^*−/−*^ mice having significantly lower REE (25%) than WT (Fig. 1F and Supplementary Table 3). In contrast, no genotype effect was observed on food consumed within 24 h or spontaneous locomotor activity (Supplementary Table 3). Next, we performed cosinor analysis to interrogate whether 24 h rhythms in metabolic cage parameters differed with respect to genotype. This analysis revealed that for energy expenditure, mesor was significantly decreased in both male (*p* = 0.0003) and female (*p* = 0.008) *Sdc4*^*−/−*^ mice (Supplementary Table 4). Interestingly, amplitude (*p* = 0.03) and acrophase (*p* = 0.008) for 24 h rhythms in respiratory exchange ratio (RER) were also significantly different between male *Sdc4*^*−/−*^ mice and WT (Fig. 1G and Supplementary Table 4); however; these parameters did not differ with respect to genotype in female mice. The genotype did not affect 24 h rhythms in activity, in either male or female mice (Supplementary Table 4).

**Fig. 2:**
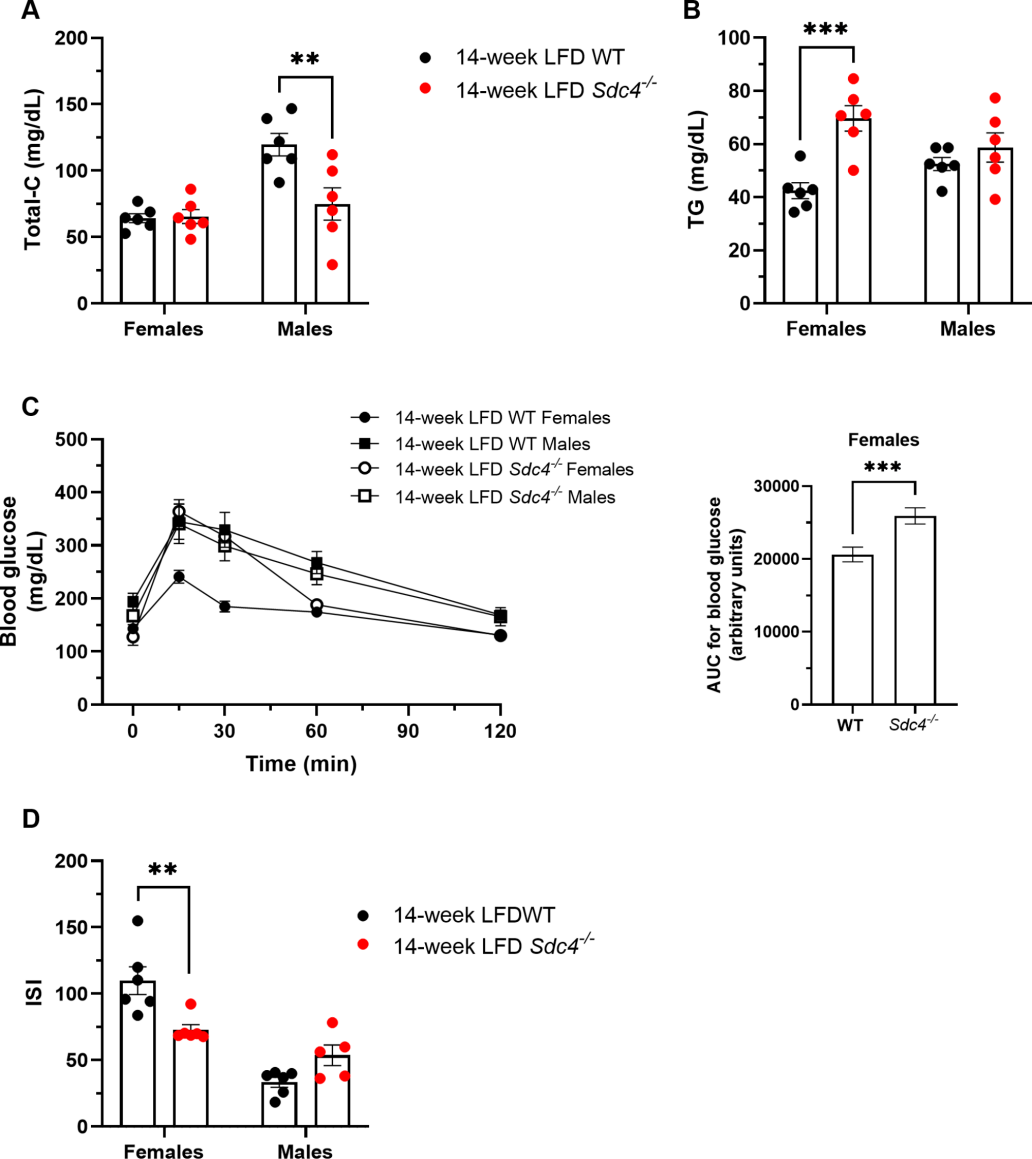
Sex-dependent alterations in cardiometabolic risk parameters of *Sdc4* deficient mice fed a 14-week LFD. **(A-D)** Following the dietary intervention, male *Sdc4*^*−/−*^ mice had lower total cholesterol (panel A), but female *Sdc4*^*−/−*^ mice had higher levels of fasting TG (panel B), reduced glucose tolerance (panel C), and lower whole-body insulin sensitivity index (panel D) than their corresponding WT mice. In panels A, B, and D, box and whiskers plots denote individual data points separated by a line representing the group median. Each individual value is plotted as a dot superimposed on the boxplots. In panel C, data indicate mean ± SE of glucose during the OGTT (left) and total area under the curve (AUC) (right) for *n* = 6–7 independent replicates. In all panels, **p < 0.01 and ***p < 0.001, obtained from Tukey *post hoc* tests for multiple comparisons

To further investigate the metabolic effects of the *Sdc4* mutation in LFD-fed mice, we next measured selected fasting plasma metabolic parameters. There was no genotype effect on fasting glucose or insulin levels (Supplementary Table 4). On the other hand, there were significant genotype-by-sex interaction effects on TC (F_1,20_=8.11, *p* = 0.0099), TG (F_1,20_=6.52, *p* = 0.0188), OGTT (F_4,800_=7.81, *p* < 0.0001), and whole-body ISI (F_1,19_=16.76, *p* = 0.0006) (Supplementary Tables 4 and 5). While LFD-fed male *Sdc4*^*−/−*^ had statistically significant lower TC levels (37%) (Fig. 2A), female *Sdc4*^*−/−*^ mice had higher (39%) levels of fasting TG (Fig. 2B), lower glucose tolerance (Fig. 2C), and reduced ISI (Fig. 2D) compared to WT.

Given that the female-specific effects of the mutation are the same of those seen in female mutant mice kept on a HFD [20], our results strongly suggest that *Sdc4* deficiency promotes increased fasting TG and insulin resistance in female mice independent of fat content in the diet.

### Genetic associations of *SDC4* SNPs with fasting TG and MetS in older people

Next, we explored genetic associations of *SDC4* SNPs with MetS in a cohort of Italian older subjects. The demographic, anthropometric, and clinical characteristics of the subjects are presented in Table 1. As expected, participants with MetS (MetS^+^) had a greater number of adverse risk factors than participants without MetS (MetS^−^). There were no significant differences in age or sex distributions between the two groups. No evidence of departure from Hardy-Weinberg equilibrium was observed for any SNPs of *SDC4* genotyped (all *p*-values > 0.05).

**Table 1:** Anthropometric and metabolic characteristics of the study cohort stratified according to absence/presence of Metabolic syndrome (MetS^−^/MetS^+^).

Characteristics	MetS^−^ (N = 161)	MetS^+^ (N = 113)	*p-value*
***Demographic factors***			
Age, Mean ± SD	73.51 ± 5.62	73.73 ± 5.81	ns
Sex, male %	54%	54%	ns
***Anthropometric parameters, Mean ± SD***			
Body mass index, kg/m^2^	25.55 ± 3.86	29.85 ± 3.36	< 0.001
Waist circumference, cm	90.38 ± 11.25	102.90 ± 10.29	< 0.001
Hip circumference, cm	100.33 ± 9.81	111.0 ± 11.02	< 0.001
Waist-to-Hip ratio	0.89 ± 0.062	0.92 ± 0.098	0.15
***Clinical parameters, Mean ± SD***			
Triglycerides, mg/dl	113.68 ± 50.26	143.42 ± 58.35	< 0.001
HDL-cholesterol, mg/dL	60.51 ± 13.88	50.63 ± 14.07	< 0.001
LDL cholesterol, mg/dl	124.39 ± 34.87	119.05 ± 35.95	ns
Total cholesterol, mg/dl	207.63 ± 39.60	198.39 ± 41.87	ns
Fasting plasma glucose, mg/dL	102.85 ± 22.67	124.89 ± 43.62	< 0.001
Glycated hemoglobin, %	5.47 ± 0.76	5.94 ± 1.02	< 0.001
Triglyceride-glucose (TyG) index	4.63 ± 0.21	4.84 ± 0.21	< 0.001
Systolic blood pressure, mmHg	132.83 ± 16.23	137.38 ± 14.01	0.018
Diastolic blood pressure, mmHg	75.77 ± 8.53	76.87 ± 8.99	ns

Table 2 reports the results of the logistic regression analysis and presents the best model for association with MetS risk. The ORs, adjusted for sex and age of the participants, showed that three SNPs, the missense variant rs2228384-T/C and the intronic variants rs2072785-G/T and rs1981429-T/G were associated with MetS. For rs2228384 and rs2072785, the recessive genetic model (homozygous for the minor allele versus all other genotypes) was the best-fitting model, and for both variants the direction of the effect was positive, corresponding to a decreased disease risk (OR = 0.34; 95% CI 0.15–0.78; *p* = 0.0069 and OR = 0.36; 95% CI 0.16–0.79; *p* = 0.0072 for rs2228384 and rs2072785, respectively). On the other hand, individuals carrying at least one copy of the minor G allele of rs1981429 (dominant genetic model) were at higher risk of MetS as compared to the group of non-carriers (OR 1.83; 95% CI 1.06–3.14; *p* = 0.027).

**Table 2:** Association analysis of selected *SDC4* SNPs with Metabolic Syndrome

SNP ID	Position (GRCh38.p14)	Genomic Location	MAF	OR (95% CI)^a,b^	*p*-values
rs2228384-T/C	45,348,351	Exon 1 (F-L)	0.45	**0.34 (0.15–0.78)** ^**R**^	**0.007**
rs2072785-G/T	45,347,894	Intron 1	0.45	**0.36 (0.16–0.79)** ^**R**^	**0.007**
rs1981429-T/G	45,347,053	Intron 1	0.40	**1.83 (1.06–3.14)** ^**D**^	**0.027**
rs2267871-A/T	45,343,730	Intron 1	0.31	1.30 (0.63–2.70)^R^	0.480
rs2251252-G/A	45,342,717	Intron 1	0.49	0.59 (0.33–1.05)^D^	0.071
rs2284277-G/A	45,338,670	Intron 1	0.22	0.87 (0.50–1.51)^D^	0.610
rs6130811-C/G	45,334,849	Intron 2	0.33	0.49 (0.17–1.41)^R^	0.170
rs2070639-T/G	45,330,210	Intron 4	0.47	1.53 (0.82–2.85)^R^	0.180
rs4599-A/G	45,325,767	3’UTR	0.21	0.71 (0.212.42)^R^	0.570
rs749111-A/T	45,322,906	3’ near gene	0.15	1.44 (0.81–2.55)^D^	0.210
rs11697824-G/A	45,322,360	3’ near gene	0.17	0.74 (0.40–1.34)^D^	0.320

Abbreviations: MAF: Minor Allele Frequency in our control group; OR: odds ratio; CI: confidence interval

^a^ Logistic regression OR adjusted for age and sex

^b^For each SNP the best model was considered: R recessive, D dominant

Significant *p*-values (< 0.05) are highlighted in bold

To explore the relationship and the possible independent effect of the significant variants, we assessed LD among them. We found a high degree of LD between rs2228384 and rs2072785 (r^2^ = 0.97), which, however, share a modest degree of LD with rs1981429 (r^2^ = 0.62), indicating that the associations might not be statistically independent. Haplotype analysis including the three SNPs showed that four out of the eight possible haplotypes were common in our study samples, whereas the remaining four haplotypes were extremely rare (Table 3). The global *p*-value for the estimated haplotypes was 0.02. We observed that individuals with haplotype T-G-G (formed by the risk alleles of the three SNPs) have a greater susceptibility to MetS (OR = 1.54, 95% CI = 1.06–2.24, *p* = 0.023), while individuals with haplotype C-T-T (formed by the protective alleles of the three SNPs) have a lower susceptibility (OR = 0.55, 95% CI = 0.30–0.83, *p* = 0.004). Thus, the haplotype analysis confirmed the results with the single markers.

**Table 3:** Association of the haplotypes derived from the rs2228384, rs2072785, and rs1981429 with Metabolic syndrome

Haplotypes			Frequencies			
rs2228384	rs2072785	rs1981429	MetS^−^	MetS^+^	OR (95% CI)	*p*-value
T	G	G	0.372	0.494	1.54 (1.06–2.24)	0.023
C	T	T	0.427	0.318	0.55 (0.3–0.83)	0.004
T	G	T	0.163	0.169	0.82 (0.49–1.39)	0.47
T	T	T	0.007	0.015	1.28 (0.27–6.05)	0.76

We next assessed whether the three SNPs might be associated with inter-individual variability in metabolic components of the MetS using linear regression model analysis adjusted for age and sex. There were no significant differences between the genotype distribution and metabolic components of MetS, except for fasting levels of TG. As shown in Table 4, we found that TG levels were significantly lower in individuals carrying at least one copy of the minor allele of rs2228384 and rs2072785 than in those homozygous for the common alleles (*β* = -0.043, *p* = 0.012 for rs2228384 and *β* = -0.037, *p* = 0.024 for rs2072785). Conversely, carriers of at least one copy of the minor allele of rs1981429 exhibited higher levels of TG (*β* = 0.038, *p* = 0.009). Notably, sex-specific analyses detected a significant association between presence of the rs1981429-G minor allele and increased levels of TG in the female cohort (*β* = 0.053, *p* = 0.008) but not in males.

**Table 4:** Association of *SDC4* SNPs with fasting TG levels and TyG index

	*Genotypes*			*Regression analysis*		
**rs228384**	**TT**	**TC**	**CC**	**β**	***SE***	***p*** ^***a***^
TG, mg/dl	140.2 ± 64	125.8 ± 52.2	112.9 ± 54.4	-0.043	0.017	0.012
TyG index	4.76 ± 0.25	4.71 ± 0.22	4.67 ± 0.23	-0.049	0.022	0.031
**rs2072785**	**GG**	**GT**	**TT**			
TG, mg/dl	139.5 ± 65.6	126.1 ± 51.4	114.4 ± 52.8	-0.037	0.016	0.024
TyG index	4.75 ± 0.24	4.70 ± 0.22	4.66 ± 0.22	-0.043	0.022	0.005
**rs1981429**	**TT**	**TG**	**GG**			
TG, mg/dl	115.2 ± 50.5	128.4 ± 49.7	142.24 ± 71	0.038	0.015	0.009
TyG index	4.66 ± 0.22	4.73 ± 0.22	4.78 ± 0.25	0.046	0.02	0.019

Data are presented as means ± SD. TG: triglycerides; TyG index: Triglyceride-Glucose index; β: unstandardized regression coefficient; SE: standard error

^a^ Adjusted for age and sex

Based on the findings in mice, we also tested genetic associations of the above SNPs with triglyceride glucose (TyG) index, a proxy of insulin resistance. In agreement with what we observed for TG levels, individuals carrying the minor allele of rs2228384-C and rs2072785-T had a lower TyG index (*β* = -0.049, *p* = 0.031 and *β* = -0.043, *p* = 0.05, respectively), while the presence of the minor allele G in rs1981429 was associated with a higher TyG index (*β* = 0.044, *p* = 0.023) (Table 4). Upon sex stratification, again the minor allele rs1981429-G associated with a higher TyG index only in females (*β* = 0.057, *p* = 0.041).

## Discussion

SDC4 is a well-recognized mechanosensor with a pivotal role in mechanotransduction at the level of the cell-matrix interface [18]. A growing body of evidence argues for a role of mechanotransduction signaling pathways in metabolic dysfunction [38, 39]. To this end, we previously reported that HFD-fed female mice lacking functional SDC4 had increased adiposity and macrophage infiltration in the visceral adipose tissue than WT mice [20]. Additionally, they had a worse metabolic profile, with higher levels of fasting plasma glucose, TC, and TG, reduced whole body ISI, as well as increased hepatic TG. In contrast, no differences were observed between HFD-fed male *Sdc4*^*−/−*^ mice and WT [20]. In this study, we report that female *Sdc4*^*−/−*^ mice fed a diet containing a lower content of fat (LFD 10% kilocalories vs. HFD 60% kilocalories), but a higher content of carbohydrates (LFD 70% kilocalories vs. HFD 20% kilocalories), displayed also higher TG and reduced whole-body insulin sensitivity than WT despite no differences in fat mass. On the other hand, LFD-fed male *Sdc4*^*−/−*^ mice had lower %FBM, smaller epididymal adipocyte size, and reduced total cholesterol. Our findings agree with quantitative genetic studies in mice revealing that pleiotropic genetic effects on metabolic phenotypes associated to the MetS are different between sexes and are modified by the composition of the diet [31]. Furthermore, sex-specific effects of the *Sdc4* null mutation have also been reported in the mouse heart, with deficiency of *Sdc4* leading to higher levels of pSer473-Akt/pSer9-GSK-3b signaling and lower levels of pThr308-Akt/Akt and GLUT4 in the heart of females but not of males [40]. Sex hormones and sex differences in energy partitioning and balance play a pivotal role in body composition and metabolic homeostasis in both rodents and humans [41]; therefore, it is likely that they are behind the observed sexually dimorphic effects of the *Sdc4* mutation. Altogether, our findings motivate additional work to define the mechanisms through which changes in SDC4 levels affect adiposity and metabolic outcomes in a sex- and diet-specific manner.

One additional result of our study is a significant reduction in fat- and lean-adjusted REE in LFD-fed male *Sdc4*^*−/−*^ mice compared to WT mice. This agrees with our earlier study in humans showing that *SDC4* rs4599 was associated with REE in American peripubertal children, with children homozygous for the major rs4599-T allele having lower REE than those heterozygous or homozygous for the minor allele [25]. Notably, the *SDC4 *rs4599 was also found linked to duration of sleep at night in the same cohort of children and those homozygous for the major rs4599-T allele and heterozygous slept longer [25]. Insufficient sleep and circadian misalignment predispose to obesity and poor metabolic health in both humans [42] and mice [43]; however, the underlying mechanisms remain unclear. We did not assess sleep/wake cycle in our mouse study, but together our results suggest that SDC4 might be involved in the control of cellular circadian oscillators. This idea is corroborated by our finding that 24-hr fluctuations in RER (e.g. metabolic fuel utilization) were significantly different between male *Sdc4*^*−/−*^ and WT mice. If the male *Sdc4*^*−/−*^ mice did in fact have better sleep duration and quality, it might explain why they were resistant to weight gain during the 14-week LFD intervention and did not show increased adiposity under HFD as previously reported [20].

Here, we also report that *SDC4* variants contribute to the risk of MetS in humans. Specifically, we showed that the minor alleles of SNPs rs2228384 (allele C) and rs2072785 (allele T) were significantly associated with reduced risk of MetS. On the other hand, an increased risk of developing the syndrome was observed for carriers of the minor allele of rs1981429 (allele G). Both protective alleles were also found linked to lower levels of fasting TG and TyG index, a biochemical marker of insulin resistance, while the opposite was observed for the MetS-risk G-allele of rs1981429. Additionally, as predicted by the mouse study, a sex-stratified analysis showed that the association between the *SDC4* rs1981429 G-allele and higher TG and TyG index was statistically significant only in females. TG levels correlate positively with insulin resistance in nondiabetic individuals [44] and high fasting TG are normally driven by increased VLDL-TG secretion and/or compromised hydrolysis of TG by LPL [45]. Earlier work demonstrated that SDC1 mediates hepatic clearance of triglyceride-rich lipoproteins in mice [46]. More recently, De Nardo and colleagues [22] also reported that overexpression of SCD4 specifically in the mouse liver results in reduced hepatic steatosis. Thus, it is likely that the genetic associations between *SDC4* and fasting TG could be due to a pivotal role for SDC4 in lipid metabolism in the human liver. The reason that changes in circulating TG were observed only in females is still unknown and needs to be further investigated.

One limitation of this study is that we did not perform experiments to determine whether the three MetS-associated SNPs may influence the function of SDC4. However, after investigating their potential functional impact by using the Genotype-Tissue Expression (GTEx) portal [47], we retrieved that SNPs rs2072785 and rs1981429, which both map in the first intron of the *SDC4* gene, had high likelihood scores of affecting *SDC4* gene expression in brain-cerebellum. While the less common allele of rs1981429 was found to decrease the expression of *SDC4* [Normalized Effect Size (NES) = 0.28, p = 8.8e-13)], the less common allele of rs2072785 was found to increase it (NES = 0.23, *p* < 1.3e-8). The cerebellum harbors a circadian clock that has been reported to be involved in anticipation of mealtime in mice [48]. Thus, our observation is intriguing and supports the idea of SDC4 as a potential regulator of cellular circadian clocks.

## Conclusions

In conclusion, together with findings from our previous research [20], the results of this study show that deletion of *Sdc4* in mice promotes substantial changes in body composition and metabolic phenotypes in a sex- and diet -specific manner. However, it is also evident from our studies in mice that the effects of the *Sdc4* deficiency on fasting TG and insulin resistance although specific to females are independent of the dietary composition. The sexually-dimorphic effect appears to take place also in humans. In this study, we found not only that *SDC4* genetic variants contribute to the risk of MetS in humans, but also that the association of *SDC4* rs1981429 with fasting TG and TyG was significant only in women when sex-stratified analyses were run. Our results, if replicated in a larger sample with a longitudinal design, could have important clinical implications. Screening for the *SDC4* variants could provide important prognostic information on the individual’s risk to develop MetS, particularly in women, and thereby help clinicians in healthcare decision-making.

### Electronic supplementary material

Below is the link to the electronic supplementary material.

Supplementary Material 1

## Data Availability

The datasets used and/or analyzed during the current study are available from the corresponding authors on reasonable request.

## References

[1] Grundy SM (2016). Metabolic syndrome update. Trends Cardiovasc Med.

[2] Lemieux I, Despres JP. Metabolic syndrome: past, Present and Future. Nutrients 2020, 12(11). 10.3390/nu12113501 PMC7696383 33202550

[3] Laaksonen DE, Lakka HM, Niskanen LK, Kaplan GA, Salonen JT, Lakka TA (2002). Metabolic syndrome and development of diabetes mellitus: application and validation of recently suggested definitions of the metabolic syndrome in a prospective cohort study. Am J Epidemiol.

[4] Mottillo S, Filion KB, Genest J, Joseph L, Pilote L, Poirier P, Rinfret S, Schiffrin EL, Eisenberg MJ (2010). The metabolic syndrome and cardiovascular risk a systematic review and meta-analysis. J Am Coll Cardiol.

[5] Lakka HM, Laaksonen DE, Lakka TA, Niskanen LK, Kumpusalo E, Tuomilehto J, Salonen JT (2002). The metabolic syndrome and total and cardiovascular disease mortality in middle-aged men. JAMA.

[6] Tirandi A, Carbone F, Montecucco F, Liberale L (2022). The role of metabolic syndrome in sudden cardiac death risk: recent evidence and future directions. Eur J Clin Invest.

[7] Noubiap JJ, Nansseu JR, Lontchi-Yimagou E, Nkeck JR, Nyaga UF, Ngouo AT, Tounouga DN, Tianyi FL, Foka AJ, Ndoadoumgue AL (2022). Geographic distribution of metabolic syndrome and its components in the general adult population: a meta-analysis of global data from 28 million individuals. Diabetes Res Clin Pract.

[8] Saklayen MG (2018). The global epidemic of the metabolic syndrome. Curr Hypertens Rep.

[9] Chakravarti R, Adams JC (2006). Comparative genomics of the syndecans defines an ancestral genomic context associated with matrilins in vertebrates. BMC Genomics.

[10] Arokiasamy S, Balderstone MJM, De Rossi G, Whiteford JR (2019). Syndecan-3 in inflammation and angiogenesis. Front Immunol.

[11] Gopal S, Arokiasamy S, Pataki C, Whiteford JR, Couchman JR (2021). Syndecan receptors: pericellular regulators in development and inflammatory disease. Open Biol.

[12] Leonova EI, Galzitskaya OV (2015). Role of Syndecans in lipid metabolism and human Diseases. Adv Exp Med Biol.

[13] Lunde IG, Herum KM, Carlson CC, Christensen G (2016). Syndecans in heart fibrosis. Cell Tissue Res.

[14] Echtermeyer F, Baciu PC, Saoncella S, Ge Y, Goetinck PF (1999). Syndecan-4 core protein is sufficient for the assembly of focal adhesions and actin stress fibers. J Cell Sci.

[15] Reizes O, Goldberger O, Smith AC, Xu Z, Bernfield M, Bickel PE (2006). Insulin promotes shedding of syndecan ectodomains from 3T3-L1 adipocytes: a proposed mechanism for stabilization of extracellular lipoprotein lipase. Biochemistry.

[16] Astudillo P, Carrasco H, Larrain J (2014). Syndecan-4 inhibits Wnt/beta-catenin signaling through regulation of low-density-lipoprotein receptor-related protein (LRP6) and R-spondin 3. Int J Biochem Cell Biol.

[17] Lambaerts K, Wilcox-Adelman SA, Zimmermann P (2009). The signaling mechanisms of syndecan heparan sulfate proteoglycans. Curr Opin Cell Biol.

[18] Chronopoulos A, Thorpe SD, Cortes E, Lachowski D, Rice AJ, Mykuliak VV, Rog T, Lee DA, Hytonen VP (2020). Del Rio Hernandez AE: Syndecan-4 tunes cell mechanics by activating the kindlin-integrin-RhoA pathway. Nat Mater.

[19] Partovian C, Ju R, Zhuang ZW, Martin KA, Simons M (2008). Syndecan-4 regulates subcellular localization of mTOR Complex2 and akt activation in a PKCalpha-dependent manner in endothelial cells. Mol Cell.

[20] De Luca M, Vecchie D, Athmanathan B, Gopalkrishna S, Valcin JA, Swain TM, Sertie R, Wekesa K, Rowe GC, Bailey SM et al. Genetic deletion of Syndecan-4 alters body composition, metabolic phenotypes, and the function of metabolic tissues in female mice Fed A High-Fat Diet. Nutrients 2019, 11(11). 10.3390/nu11112810 PMC6893658 31752080

[21] Xia SJ, Tang LZ, Li WH, Xu ZS, Zhang LL, Cheng FG, Chen HX, Wang ZH, Luo YC, Dai AN (2021). Serum syndecan-4 is associated with nonalcoholic fatty liver disease. J Dig Dis.

[22] De Nardo W, Miotto PM, Bayliss J, Nie S, Keenan SN, Montgomery MK, Watt MJ (2022). Proteomic analysis reveals exercise training induced remodelling of hepatokine secretion and uncovers syndecan-4 as a regulator of hepatic lipid metabolism. Mol Metab.

[23] Herum KM, Romaine A, Wang A, Melleby AO, Strand ME, Pacheco J, Braathen B, Duner P, Tonnessen T, Lunde IG (2020). Syndecan-4 protects the heart from the Profibrotic Effects of Thrombin-Cleaved Osteopontin. J Am Heart Assoc.

[24] Cornelison DD, Wilcox-Adelman SA, Goetinck PF, Rauvala H, Rapraeger AC, Olwin BB (2004). Essential and separable roles for Syndecan-3 and Syndecan-4 in skeletal muscle development and regeneration. Genes Dev.

[25] De Luca M, Klimentidis YC, Casazza K, Chambers MM, Cho R, Harbison ST, Jumbo-Lucioni P, Zhang S, Leips J, Fernandez JR (2010). A conserved role for syndecan family members in the regulation of whole-body energy metabolism. PLoS ONE.

[26] Rose G, Crocco P, De Rango F, Corsonello A, Lattanzio F, De Luca M, Passarino G (2015). Metabolism and successful aging: polymorphic variation of syndecan-4 (SDC4) gene associate with longevity and lipid profile in healthy elderly italian subjects. Mech Ageing Dev.

[27] Kunnas T, Nikkari ST (2014). Contribution of syndecan-4 genetic variants to hypertension, the TAMRISK study. BMC Res Notes.

[28] Ordovas JM, Shen J (2008). Gene-environment interactions and susceptibility to metabolic syndrome and other chronic diseases. J Periodontol.

[29] Menotti A, Keys A, Blackburn H, Karvonen M, Punsar S, Nissinen A, Pekkanen J, Kromhout D, Giampaoli S, Seccareccia F (1991). Blood pressure changes as predictors of future mortality in the seven countries study. J Hum Hypertens.

[30] Menotti A, Keys A, Kromhout D, Nissinen A, Blackburn H, Fidanza F, Giampaoli S, Karvonen M, Pekkanen J, Punsar S (1991). All cause mortality and its determinants in middle aged men in Finland, the Netherlands, and Italy in a 25 year follow up. J Epidemiol Community Health.

[31] Lawson HA, Cady JE, Partridge C, Wolf JB, Semenkovich CF, Cheverud JM (2011). Genetic effects at pleiotropic loci are context-dependent with consequences for the maintenance of genetic variation in populations. PLoS Genet.

[32] Matsuda M, DeFronzo RA (1999). Insulin sensitivity indices obtained from oral glucose tolerance testing: comparison with the euglycemic insulin clamp. Diabetes Care.

[33] Peliciari-Garcia RA, Bargi-Souza P, Young ME, Nunes MT (2018). Repercussions of hypo and hyperthyroidism on the heart circadian clock. Chronobiol Int.

[34] Simental-Mendia LE, Rodriguez-Moran M, Guerrero-Romero F (2008). The product of fasting glucose and triglycerides as surrogate for identifying insulin resistance in apparently healthy subjects. Metab Syndr Relat Disord.

[35] Expert Panel on Detection E (2001). Treatment of high blood cholesterol in A: executive summary of the third report of the national cholesterol Education Program (NCEP) Expert Panel on detection, evaluation, and treatment of high blood cholesterol in adults (Adult Treatment Panel III). JAMA.

[36] Shi YY, He L (2005). SHEsis, a powerful software platform for analyses of linkage disequilibrium, haplotype construction, and genetic association at polymorphism loci. Cell Res.

[37] Machiela MJ, Chanock SJ (2015). LDlink: a web-based application for exploring population-specific haplotype structure and linking correlated alleles of possible functional variants. Bioinformatics.

[38] Liao X, Li X, Liu R (2023). Extracellular-matrix mechanics regulate cellular metabolism: a ninja warrior behind mechano-chemo signaling crosstalk. Rev Endocr Metab Disord.

[39] Salvi AM, DeMali KA (2018). Mechanisms linking mechanotransduction and cell metabolism. Curr Opin Cell Biol.

[40] Stole TP, Lunde M, Shen X, Martinsen M, Lunde PK, Li J, Lockwood F, Sjaastad I, Louch WE, Aronsen JM (2022). The female syndecan-4(-/-) heart has smaller cardiomyocytes, augmented insulin/pSer473-Akt/pSer9-GSK-3beta signaling, and lowered SCOP, pThr308-Akt/Akt and GLUT4 levels. Front Cell Dev Biol.

[41] Mauvais-Jarvis F (2015). Sex differences in metabolic homeostasis, diabetes, and obesity. Biol Sex Differ.

[42] Chaput JP, McHill AW, Cox RC, Broussard JL, Dutil C, da Costa BGG, Sampasa-Kanyinga H, Wright KP (2023). The role of insufficient sleep and circadian misalignment in obesity. Nat Rev Endocrinol.

[43] Mavanji V, Billington CJ, Kotz CM, Teske JA (2012). Sleep and obesity: a focus on animal models. Neurosci Biobehav Rev.

[44] Ma M, Liu H, Yu J, He S, Li P, Ma C, Zhang H, Xu L, Ping F, Li W (2020). Triglyceride is independently correlated with insulin resistance and islet beta cell function: a study in population with different glucose and lipid metabolism states. Lipids Health Dis.

[45] Keirns BH, Sciarrillo CM, Koemel NA, Emerson SR (2021). Fasting, non-fasting and postprandial triglycerides for screening cardiometabolic risk. J Nutr Sci.

[46] Stanford KI, Bishop JR, Foley EM, Gonzales JC, Niesman IR, Witztum JL, Esko JD (2009). Syndecan-1 is the primary heparan sulfate proteoglycan mediating hepatic clearance of triglyceride-rich lipoproteins in mice. J Clin Invest.

[47] Consortium GT (2015). Human genomics. The genotype-tissue expression (GTEx) pilot analysis: multitissue gene regulation in humans. Science.

[48] Mendoza J, Pevet P, Felder-Schmittbuhl MP, Bailly Y, Challet E (2010). The cerebellum harbors a circadian oscillator involved in food anticipation. J Neurosci.

