# Correction: Syndecan-4 as a genetic determinant of the metabolic syndrome

**DOI:** 10.1186/s13098-023-01140-8

**Published:** 2023-08-03

**Authors:** Paolina Crocco, Denise Vecchie, Sreejit Gopalkrishna, Serena Dato, Giuseppe Passarino, Martin E. Young, Prabhakara R. Nagareddy, Giuseppina Rose, Maria De Luca

**Affiliations:** 1 Department of Biology, Ecology, and Earth Sciences, University of Calabria, Rende, 87036 Italy; 2 Department of Nutrition Sciences, University of Alabama at Birmingham, Birmingham, AL, 35294 USA; 3 Department of Surgery, Division of Cardiac Surgery, The Ohio State University Wexner Medical Center, Columbus, OH, USA; 4 Division of Cardiovascular Diseases, Department of Medicine, University of Alabama at Birmingham, Birmingham, AL, 35294 USA

**Correction: Diabetol Metab Syndr (2023) 15:156**

10.1186/s13098-023-01132-8

Following publication of the original article [1], the author identified an error in the affiliation of Maria De Luca.

The correct affiliation of Maria De Luca is Department of Nutrition Sciences, University of Alabama at Birmingham, Birmingham, AL, 35294, USA.

The affiliation has been updated and the original article [1] has been corrected.

## References

[1] Crocco P, Vecchie D, Gopalkrishna S, et al. Syndecan-4 as a genetic determinant of the metabolic syndrome. Diabetol Metab Syndr. 2023;15:156. 10.1186/s13098-023-01132-8 10.1186/s13098-023-01132-8 PMC10351106 37461091

