# Supplementary material for: Syndecan-4 as a genetic determinant of the metabolic syndrome
Source: Diabetol Metab Syndr. 2023 Jul 17;15:156. doi: 10.1186/s13098-023-01132-8 (PMC10351106; doi:10.1186/s13098-023-01132-8)
Supplement: Supplementary file 1 — Supplementary Material 1 [file 13098_2023_1132_MOESM1_ESM.docx]

**Supplemental Material**

**Table S1: Three-way ANOVA for percentage body weight changes in female and male *Sdc4^-/-^* and WT mice fed a LFD for 14 weeks.**

| ***Source of Variation*** | ***df*** ^a^ | ***MS***^b^ | ***F*** | ***P-value*** |
| --- | --- | --- | --- | --- |
| Time | 10 | 1702 | 52.15 | <0.0001 |
| Sex | 1 | 15803 | 86.95 | <0.0001 |
| Genotype | 1 | 2.781 | 0.02 | 0.9026 |
| Time*Sex | 10 | 617.9 | 18.94 | <0.0001 |
| Time*Genotype | 10 | 52.80 | 1.62 | 0.1024 |
| Sex*Genotype | 1 | 2404 | 13.22 | 0.0014 |
| Time*Genotype*Sex | 10 | 163.5 | 5.01 | <0.0001 |
| Error | 230 | 32.63 |  |  |

^a^Degrees of freedom. ^b^Mean Squares computed from Type III Sums of Squares.

**Table S2: Two-way ANOVA for %FBM and %LBM in female and male *Sdc4^-/-^* and WT mice fed a LFD for 14 weeks.**

| **Phenotype** | ***Source of Variation*** | ***df*** ^a^ | ***MS***^b^ | ***F*** | ***P-value*** |
| --- | --- | --- | --- | --- | --- |
| %FBM | Sex | 1 | 244.5 | 30.71 | <.0001 |
|  | Genotype | 1 | 61.51 | 7.73 | 0.0107 |
|  | Genotype *Sex | 1 | 74.18 | 9.32 | 0.0056 |
|  | Error | 23 | 7.961 |  |  |
|  |  |  |  |  |  |
| %LBM | Sex | 1 | 168.1 | 21.75 | 0.0001 |
|  | Genotype | 1 | 36.24 | 4.69 | 0.0410 |
|  | Genotype *Sex | 1 | 79.50 | 10.29 | 0.0039 |
|  | Error | 23 | 7.729 |  |  |

^a^Degrees of freedom. ^b^Mean Squares computed from Type III Sums of Squares. %FBM: percentage fat body mass. %LBM: percentage lean body mass.

**Table S3: Two-way ANOVA/ANCOVA for food intake, REE, and locomotor activity in female and male *Sdc4^-/-^* and WT mice fed a LFD for 14 weeks.**

| **Phenotype** | ***Source of Variation*** | ***df*** ^a^ | ***MS***^b^ | ***F*** | ***P-value*** |
| --- | --- | --- | --- | --- | --- |
| Food intake | Sex | 1 | 1.614 | 1.00 | 0.1930 |
|  | Genotype | 1 | 0.065 | 0.07 | 0.7912 |
|  | Genotype*Sex | 1 | 0.178 | 0.20 | 0.6629 |
|  | Error | 23 | 0.881 |  |  |
|  |  |  |  |  |  |
| REE | Fat mass | 1 | 0.001 | 0.84 | 0.3695 |
|  | Lean mass | 1 | 0.054 | 6.65 | 0.0175 |
|  | Sex | 1 | 0.001 | 1.32 | 0.2627 |
|  | Genotype | 1 | 0.011 | 13.36 | 0.0015 |
|  | Genotype*Sex | 1 | 0.007 | 8.35 | 0.0088 |
|  | Error | 21 | 0.001 |  |  |
|  |  |  |  |  |  |
| Locomotor activity | Sex | 1 | 2.498 | 9.53 | 0.0052 |
|  | Genotype | 1 | 0.0002 | 0.00 | 0.9805 |
|  | Genotype*Sex | 1 | 0.0003 | 0.00 | 0.9705 |
|  | Error | 23 | 0.2622 |  |  |

^a^Degrees of freedom. ^b^Mean Squares computed from Type III Sums of Squares. REE: resting energy expenditure. Locomotor activity data were log_10_ transformed to fulfill the assumption of normality.

**Table S4: Cosinor analysis for metabolic cage data.**

| **Sex/Parameter** | **Mesor** | | **Amplitude** | | **Acrophase** | |
| --- | --- | --- | --- | --- | --- | --- |
| **Male** | **WT** | ***Sdc4^-/-^*** | **WT** | ***Sdc4^-/-^*** | **WT** | ***Sdc4^-/-^*** |
| **RER** | 0.875 ± 0.006 | 0.893 ± 0.007 | 0.111 ± 0.009 | 0.072 ± 0.010* | 19.13 ± 0.27 | 16.96 ± 0.49* |
| **Energy Expenditure** | 0.510 ± 0.004 | 0.457 ± 0.006* | 0.049 ± 0.006 | 0.068 ± 0.008 | 16.68 ± 0.41 | 17.28 ± 0.40 |
| **Locomotor Activity** | 154.0 ± 10.5 | 152.2 ± 11.7 | 94.7 ± 15.8 | 100.7 ± 17.6 | 16.81 ± 0.56 | 17.42 ± 0.58 |
| **Female** | **WT** | ***Sdc4^-/-^*** | **WT** | ***Sdc4^-/-^*** | **WT** | ***Sdc4^-/-^*** |
| **RER** | 0.857 ± 0.01 | 0.864 ± 0.010 | 0.099 ± 0.015 | 0.061 ± 0.015 | 17.91 ± 0.48 | 16.36 ± 0.82 |
| **Energy Expenditure** | 0.467 ± 0.008 | 0.430 ± 0.005* | 0.051 ± 0.012 | 0.052 ± 0.007 | 17.47 ± 0.75 | 17.13 ± 0.47 |
| **Locomotor Activity** | 357.7 ± 38.5 | 341.9 ± 42.4 | 248.6 ± 58.0 | 333.9 ± 64.3 | 18.92 ± 0.78 | 18.04 ± 0.63 |

REE: resting energy expenditure.* represents p<0.05 for WT versus *Sdc4^-/^*

**Table S5: Two-way ANOVA for metabolic parameters in female and male *Sdc4^-/-^* and WT mice fed a LFD for 14 weeks.**

| **Phenotype** | ***Source of Variation*** | ***df*** ^a^ | ***MS***^b^ | ***F*** | ***P-value*** |
| --- | --- | --- | --- | --- | --- |
| Total cholesterol | Sex | 1 | 6292 | 16.20 | 0.0007 |
|  | Genotype | 1 | 2819  2819  9 | 7.26 | 0.0140 |
|  | Genotype *Sex | 1 | 3149 | 8.11 | 0.0099 |
|  | Error | 20 | 388.3 |  |  |
|  |  |  |  |  |  |
| Triglycerides | Sex | 1 | 1.242 | 0.01 | 0.9132 |
|  | Genotype | 1 | 1676 | 16.38 | 0.0006 |
|  | Genotype *Sex | 1 | 668.4 | 6.52 | 0.0188 |
|  | Error | 20 | 102.3 |  |  |
|  |  |  |  |  |  |
| Insulin | Sex | 1 | 0.228 | 6.91 | 0.0161 |
|  | Genotype | 1 | 0.073 | 2.20 | 0.1536 |
|  | Genotype *Sex | 1 | 0.023 | 0.69 | 0.4155 |
|  | Error | 20 | 0.033 |  |  |
|  |  |  |  |  |  |
| Glucose | Sex | 1 | 12467 | 10.50 | 0.0041 |
|  | Genotype | 1 | 2752 | 2.32 | 0.1435 |
|  | Genotype *Sex | 1 | 198.4 | 0.17 | 0.6870 |
|  | Error | 20 | 1187 |  |  |
|  |  |  |  |  |  |
| Insulin sensitivity index | Sex | 1 | 13069 | 46.78 | <.0001 |
|  | Genotype | 1 | 390.9 | 1.40 | 0.2514 |
|  | Genotype *Sex | 1 | 4683 | 16.76 | 0.0006 |
|  | Error | 19 | 279.3 |  |  |

^a^Degrees of freedom. ^b^Mean Squares computed from Type III Sums of Squares. Insulin, glucose, and insulin sensitivity index data were log_10_ transformed to fulfill the assumption of normality.

**Table S6: Three-way ANOVA for OGTT in female and male *Sdc4^-/-^* and WT mice fed a LFD for 14 weeks.**

| ***Source of Variation*** | ***df*** ^a^ | ***MS***^b^ | ***F*** | ***P-value*** |
| --- | --- | --- | --- | --- |
| Time | 4 | 138771 | 137.20 | <0.0001 |
| Sex | 1 | 82111 | 11.39 | 0.0030 |
| Genotype | 1 | 8317 | 1.15 | 0.2956 |
| Time*Sex | 4 | 1630 | 1.61 | 0.1794 |
| Time*Genotype | 4 | 7877 | 7.79 | <0.0001 |
| Sex*Genotype | 1 | 34510 | 4.79 | 0.0407 |
| Time*Genotype*Sex | 4 | 7900 | 7.81 | <0.0001 |
| Error | 80 | 1011 |  |  |

^a^Degrees of freedom. ^b^Mean Squares computed from Type III Sums of Squares. OGTT: oral glucose tolerance test.
